# Postpartum Septic Shock Due to Clostridium Perfringens From Chorioamnionitis: A Rare Case

**DOI:** 10.7759/cureus.38075

**Published:** 2023-04-24

**Authors:** Eihab A Subahi, Sagda Sayed, Abdalla Fadul, Elrazi A Ali

**Affiliations:** 1 Department of Internal Medicine, Hamad Medical Corporation, Doha, QAT; 2 Department of Psychiatry, Hamad Medical Corporation, Doha, QAT; 3 Department of Internal Medicine, One Brooklyn Health/Interfaith Medical Center, Brooklyn, USA

**Keywords:** postpartum sepsis, pregnancy, chorioamnionitis, septic shock, clostridium perfringens

## Abstract

Although postpartum infections caused by *Clostridium *species are relatively rare, they can have severe consequences if not promptly identified and treated. Clostridial uterine infections typically originate as localized chorioamnionitis, stemming from fetal and/or placental tissue infection. The infection may then spread to the uterine wall and endometrial tissues, and in the most severe cases, it may result in sepsis and shock. These infections can cause serious illness and a high mortality rate without proper treatment. We describe the case of a 26-year-old primigravida woman at 39 weeks gestation who presented with active labor. *Clostridium perfringens *was isolated from her blood culture, which led to an intrapartum fever and postpartum septic shock. The patient was admitted to the intensive care unit and received appropriate management, resulting in a favorable outcome.

## Introduction

*Clostridium perfringens*, formerly known as *Clostridium welchii*, is a rare cause of intrauterine infection. Clostridia can be isolated from the genital tract in approximately 10% of women as part of the normal vaginal microflora. These bacteria are found in up to 20% of non-sexually transmitted disease cases and may be present as a component of bacterial vaginosis [1,2].

Although uncommon, postpartum infections caused by clostridia can be severe. Clostridial uterine infections typically originate as localized chorioamnionitis due to infection of the fetal and placental tissues. The infection may then spread to the uterine wall and endometrial tissues and, in the most severe cases, result in uterine necrosis resulting in sepsis [1,2]. If not treated promptly, the toxins produced by clostridia during septicemia can lead to serious illness and devastating clinical outcomes. Recent mortality rates range from 27% to 58% and 30-day mortality can be up to 73% [3,4]. Early antibiotic and resuscitation strategies have somewhat improved the prognosis in recent years. We present a case of *Clostridium perfringens *isolated from a blood culture that led to intrapartum fever and postpartum septic shock.

## Case presentation

A 26-year-old primigravida woman at 39 weeks gestation presented with labor pain and was admitted to the labor room for active labor. Two hours after the rupture of membranes, she developed a high-grade fever with rigors and chills, along with a non-reassuring cardiotocograph, raising suspicions of chorioamnionitis. She was immediately administered ampicillin and gentamicin for the suspected chorioamnionitis and underwent a low Kiwi vacuum-assisted vaginal delivery with an episiotomy incision. The baby was delivered, and the patient experienced approximately 700 mL of blood loss due to atonic postpartum hemorrhage. She received 600 µg of misoprostol and a 30-unit intravenous (IV) oxytocin infusion.

Soon after delivery, while still in the labor room, the patient remained febrile and developed tachycardia and hypotension (blood pressure 88/54 mmHg, pulse rate 130 bpm, and temperature 38°C). She was given IV fluids (30 ml/kg ) and antibiotics (ceftriaxone and metronidazole). Her initial laboratory results showed a total white blood cell (WBC) count of 8.8 x10^3/µL, hemoglobin (Hb) 11.6 g/dL, and platelets 289 x10^3/µL. Her C-reactive protein (CRP) was elevated at 21.5 mg/L, while her procalcitonin (0.05 ng/mL) and lactic acid (1.9 mmol/L) were within reference ranges. Also, her renal and liver functions were both within reference limits.

Despite fluid resuscitation (30 ml/kg ), the patient continued to exhibit tachycardia and hypotension. Repeated laboratory tests showed an increase in WBC count to 14, then 18.6 x10^3/µL, a decrease in Hb to 8.1, then 5.8 g/dL (with no laboratory signs suggesting hemolysis), and normal platelet levels. Her international normalized ratio (INR) was 1.1, then later 1.2. Her CRP increased to 26.7, 64.0, and then 187.1 mg/L. Her procalcitonin levels rose to 7.08, 26.40, then >100.00 ng/mL, and lactic acid levels increased to 4, then 6.3 mmol/L. Her creatinine levels increased to 82, 116, and 135 µmol/L.

Due to post-delivery hemodynamic instability, worsening blood parameters, and the development of non-oliguric acute kidney injury, the patient was admitted to the intensive care unit (ICU) with a diagnosis of septic shock. She received proper fluid resuscitation, inotropic support (noradrenaline), blood transfusions, and monitoring. Antibiotic treatment was escalated to include meropenem, vancomycin, and clindamycin. The blood culture revealed *Clostridium perfringens *growth, but the placental tissue culture showed numerous polymorphonuclear cells without any organisms. The patient stabilized within 24 hours, was weaned from inotropic support, and became afebrile. Antibiotics were adjusted to penicillin G based on the antibiotic susceptibility test, and clindamycin was continued for 10 to 14 days. An abdominal ultrasound showed no intra-abdominal collections.

## Discussion

*Clostridium perfringens* is an anerobic, gram-positive, spore-forming, rod-shaped bacterium belonging to the *Clostridium* genus. Ubiquitous in nature, it can be found in decaying plants, marine sediment, the human intestinal tract, and soil. It has also been isolated in 4% of cervical cultures [5,6]. *Clostridium perfringens* produces over 20 exotoxins, with α-toxin (lecithinase C) playing a significant role in gas gangrene. This toxin inserts into the plasma membrane of cells, generating gaps in the membrane that disrupt normal cellular function. α-toxin is responsible for rapid intravascular hemolysis, causing dramatic anemia and jaundice in approximately 15% of patients with bacteremia [5]. Circulatory disintegration can also occur due to α-toxin suppressing cardiac contractility, resulting in hypotension that typically does not respond to fluid challenges [5]. In our case, the patient received appropriate fluid resuscitation but remained tachycardic and hypotensive, leading to a diagnosis of septic shock and ICU admission for inotropic support (noradrenaline). 

Intra-amniotic infection syndrome (IAIS), also known as chorioamnionitis, is a clinically detectable infection of the amniotic fluid and fetal membranes during pregnancy. Most IAIS cases originate when vaginal microorganisms ascend into the intrauterine cavity after membrane rupture. It is believed that the dissemination of Clostridium perfringens to the blood may be due to ascendant transmission from maternal bowel flora, which then passes through the placenta [2]. As our patient developed intrapartum fever two hours after membrane rupture, ascendant transmission is valid as the most common cause of clostridial sepsis.

However, most clostridial sepsis cases originate from the female genital tract, with the introduction of a foreign body often preceding the infection. Residual necrotic fetal and placental tissues and traumatized endometrium within the uterus may facilitate clostridial growth [2]. Our patient had a urinary catheter inserted before delivery and underwent a low Kiwi vacuum-assisted vaginal delivery with episiotomy incision. These maneuvers and procedures could lead to endothelial injury, facilitating bacterial entry from the genital tract to the bloodstream and causing bacteremia and sepsis.

*Clostridium perfringens* thrive in necrotic tissues with anaerobic environments, so it is important to remove the necrotic tissue required for bacterial survival. Decker and Hall argued that curettage of the endometrial cavity could leave behind necrotic tissue and potentially traumatize healthy tissue, creating more necrotic tissue [5,7]. They suggested that *Clostridium *infection associated with sepsis is an indication of hysterectomy. Other authors have proposed that *Clostridium *infection associated with hemolysis also indicates hysterectomy [8]. However, there is currently no convincing evidence that the benefits of these procedures outweigh their inherent risks [5]. Our patient experienced a significant drop in hemoglobin, but investigations were negative for hemolysis, indicating that her hemoglobin decrease may be due to atonic postpartum hemorrhage.

Although rare, *Clostridium perfringens *sepsis and severe hemolysis are extremely deadly compared to *Clostridium perfringens *bacteremia without overt hemolysis, which is relatively common [5,9]. The outcomes of more recent cases, along with ours, suggest that aggressive management with uterine and ovarian conservation may be a safe and effective option in managing severe *Clostridium *infections [9]. In the absence of hemolysis, aggressive management using antibiotics and multidisciplinary team input may allow these rare but critically ill patients the opportunity to achieve a subsequent pregnancy in the future [5,9].

## Conclusions

*Clostridium perfringens* septicemia without overt hemolysis is relatively uncommon, but the overall mortality rate is high when the infection induces severe hemolysis. Hemolysis has been considered the most extensive and adverse prognostic sign associated with the infection. In the absence of hemolysis, aggressive management with prompt administration of antibiotics, multidisciplinary team input, and conservation of the uterus and ovaries may be a safe and effective option for managing severe *Clostridium *infections.
